# Alcohol industry corporate social responsibility initiatives and harmful drinking: a systematic review

**DOI:** 10.1093/eurpub/cky065

**Published:** 2018-04-25

**Authors:** Melissa Mialon, Jim McCambridge

**Affiliations:** Department of Health Sciences, University of York

## Abstract

**Background:**

There is growing awareness of the detrimental effects of alcohol industry commercial activities, and concern about possible adverse impacts of its corporate social responsibility (CSR) initiatives, on public health. The aims of this systematic review were to summarize and examine what is known about CSR initiatives undertaken by alcohol industry actors in respect of harmful drinking globally.

**Methods:**

We searched for peer-reviewed studies published since 1980 of alcohol industry CSR initiatives in seven electronic databases. The basic search strategy was organized around the three constructs of ‘alcohol’, ‘industry’ and ‘corporate social responsibility’. We performed the searches on 21 July 2017. Data from included studies were analyzed inductively, according to the extent to which they addressed specified research objectives.

**Results:**

A total of 21 studies were included. We identified five types of CSR initiatives relevant to the reduction of harmful drinking: alcohol information and education provision; drink driving prevention; research involvement; policy involvement and the creation of social aspects organizations. Individual companies appear to undertake different CSR initiatives than do industry-funded social aspects organizations. There is no robust evidence that alcohol industry CSR initiatives reduce harmful drinking. There is good evidence, however, that CSR initiatives are used to influence the framing of the nature of alcohol-related issues in line with industry interests.

**Conclusions:**

This research literature is at an early stage of development. Alcohol policy measures to reduce harmful drinking are needed, and the alcohol industry CSR initiatives studied so far do not contribute to the attainment of this goal.

## Introduction

Today most large companies in the alcohol industry, like other corporate sectors, undertake corporate social responsibility (CSR) initiatives.^1^ Social responsibility in businesses is informed by a range of principles, including those that arise from outside the corporate sphere, such as the United Nations (UN) Global Compact,^2^ or the International Organization for Standardization (ISO).^3^ The latter details in ISO 26 000^3,^^4^ that ‘business and organizations do not operate in a vacuum. Their relationship to the society and environment in which they operate is a critical factor in their ability to continue to operate effectively’ and defines a ‘CSR initiative’ as ‘programme or activity expressly devoted to meeting a particular aim related to social responsibility’.^3^^,^^4^

Within the alcohol industry, CSR initiatives are usually directed at limiting the damage caused by alcohol^1^^,^^5^ and appear to give prominence to responsible drinking, voluntary regulation and philanthropy.^5^ In 2012, 12 global alcohol producer organizations announced their ‘wish to demonstrate their support of international efforts to improve health and social outcomes for individuals, families and communities’,^6^ through a series of commitments: reducing under-age drinking; strengthening and expanding marketing codes of practice; providing consumer information and responsible product innovation; reducing drinking and driving and enlisting the support of retailers to reduce harmful drinking.^7^

Alcohol industry-funded ‘social aspects’ organizations were created by companies in collaboration with each other as vehicles for CSR.^1^^,^^8^^,^^9^ Stichting Verantwoorde Alcoholconsumptie emerged in 1984 in the Netherlands,^9^ and the number of social aspects organizations has grown rapidly in recent years, with more than 40 organizations in existence globally in 2013.^1^ Research funding organizations, a more specific form of social aspects organization, have a somewhat older history, having been in existence for almost 50 years.^1^ Today, the major global alcohol producer companies are members of the International Alliance for Responsible Drinking (IARD), one such organization that presents itself as being ‘part of the solution envisaged by the world’s governments when they agreed to the global target of a 10% reduction in the harmful use of alcohol by 2025’.^10^

There is growing concern in the public health community about CSR in the alcohol industry,^1^^,^^5^^,^^8^ as well as in other industries.^11^^,^^12^ It has been suggested that CSR initiatives may be designed to serve public relations goals and the broader political and economic interests of alcohol industry actors.^1^^,^^5^^,^^13^ Critics of alcohol industry CSR point towards an inherent conflict of interest that exists between the economic objectives of the industry, which are concerned with maximizing profits, most of which are generated from harmful drinking, and public health objectives to reduce the harms caused by alcohol, which generally require reductions in alcohol consumption.^1^^,^^5^^,^^8^^,^^14^

Garriga and Mele^15^ identify four types of CSR theory as follows: (i) instrumental (CSR used to advance primarily economic objectives); (ii) political (CSR used to influence policy and extend power within society); (iii) integrative (where social objectives are combined with economic ones) and (iv) ethical (where responsibilities to society are met). The public health community largely regards alcohol industry CSR as instrumental and/or political.

To our knowledge, there is no systematic review of existing studies of alcohol industry CSR initiatives. Two related reviews investigated UK community-level partnership interventions,^16^ and industry self-regulation of alcohol marketing,^17^ respectively.

The aims of this systematic review were to summarize and examine what is known about CSR initiatives undertaken by alcohol industry actors globally with respect to the reduction of harmful drinking. This study had the following objectives:

- To develop a typology of alcohol industry CSR initiatives;
- To examine studied CSR initiatives in relation to: individual alcohol industry actors; types of initiative; geographical coverage; targeted populations and partners;
- To identify the evidence available on the effects of alcohol industry CSR initiatives on harmful drinking, including evaluation studies of the extent to which they fulfil their stated aims;
- To identify evidence of the political uses of CSR by alcohol industry actors (i.e. in influencing alcohol policies to reduce harmful drinking);
- To identify gaps in current evidence on CSR initiatives in the alcohol industry.

## Methods

This systematic review is reported according to the Preferred Reporting Items for Systematic Reviews and Meta-Analyses (PRISMA) guidance. For the purposes of this study, we excluded studies of:

- investment of an organization in CSR,^18^ as such studies are not designed to be informative about particular CSR initiatives, our object of study;
- any CSR component that is a part of a product promotion/advertizement or other forms of marketing *(*e.g.^19^^,^^20^), including, public relations campaigns and event sponsorships associated with particular brands. The focus of this study is initiatives which are exclusively CSR interventions, due to prior studies of CSR in marketing;
- self-regulation of marketing and/or advertizement *(*e.g.^17^^,^^21^), as this has been previously studied;
- other CSR initiatives similarly closely connected to the core economic operations of companies (serving alcohol, product and supply chain activities, employee welfare, environmental sustainability—e.g.: initiatives which focus on waste, use of water or energy efficiency). Such initiatives may be designed to reduce costs as well as address societal, environmental or political objectives.^22^^,^^23^

These decisions permitted a study focus on CSR initiatives that have no other explicit business aims and thus solely can address the reduction of harmful drinking. We developed literature search strategies using both Medical Subjects Headings terms and key words in seven databases:

- Web of Science Core Collection (Web of Science interface);
- CINAHL Plus (EBSCOhost interface);
- Business Source Premier (EBSCOhost interface);
- Embase (Ovid interface);
- MEDLINE (Ovid interface);
- PsycINFO (Ovid interface);
- Scopus (Scopus interface).

We performed the searches on 21 July 2017. We organized the basic search strategy around the three constructs of ‘alcohol’, ‘industry’ and ‘corporate social responsibility’. It was developed with the support of a specialist librarian. We present the search strategies in Supplementary material S1.

To be included in this review, studies had to meet the following inclusion criteria:

- Be published in peer-reviewed journals since 1980;
- Be published in the English language;
- Seek to study the CSR initiatives of alcohol industry organizations;
- Comprise studies presenting primary or secondary research data published as full length papers or short reports;
- Studies of CSR initiatives across multiple sectors can be included if alcohol industry data are presented separately;
- Be focused on CSR initiatives at the supra-national or national (rather than local) levels.

We excluded studies included in other systematic reviews in this series (of policy involvement and public health surveillance studies) as they do not seek to study CSR. We chose the year 1980 as sufficiently early to capture data on contemporary CSR, predating the global concentration of alcohol producers since the 1990s and the emergence of social aspects organizations.^9^

MM screened titles and abstracts. Both authors assessed full text papers retrieved for inclusion in this review. We downloaded and imported the retrieved material, and removed duplicates, using EndNote. MM hand searched the journal Addiction and conducted backwards and forwards citation searches, using the references cited in included data sources, and Web of Science, respectively. We contacted topic experts to request additional studies that we may have missed. Both reviewers determined eligibility separately. Any disagreements were resolved through discussion. MM extracted data from the publications, using cutting and past into an Excel document, as well as NVivo.

We structured our approach to data analysis using the research objectives, following both authors reading of all included papers and discussing how to proceed. For objectives one and two, MM extracted and coded data in a first stage, with both authors refining the coding through discussions in later stages in the analysis. These findings are largely tabulated. For objectives three and four, both authors extracted data into NVivo and Word, which was coded as far as possible inductively within each research objective. JM examined the methodological characteristics of the included studies. We provide a narrative synthesis of these findings, drawing on studies in light of the content of their findings and the methods used. We did not publish a protocol for this review.

## Results

**Figure 1:**
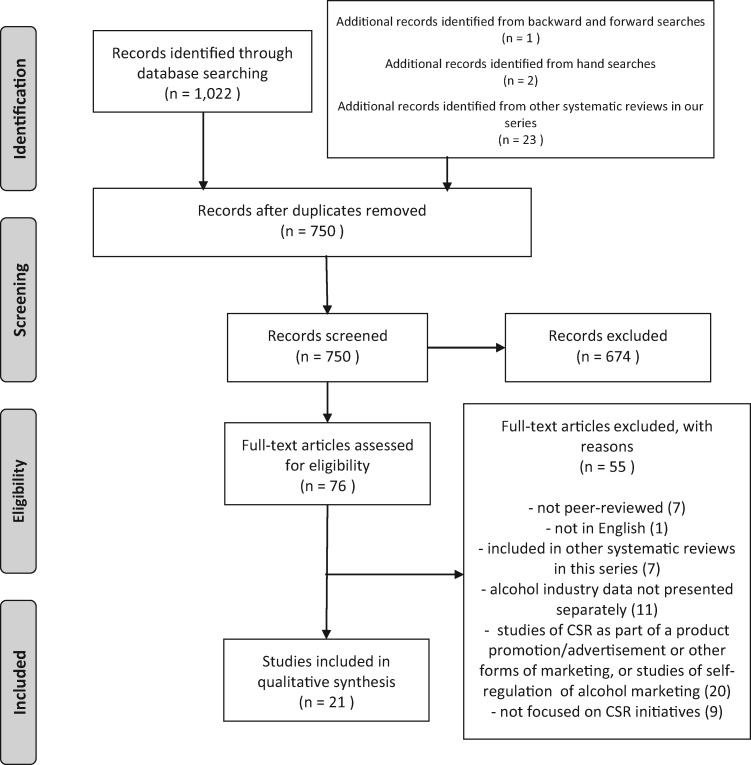
PRISMA flow diagram

We provide details of the data collection process in the PRISMA flowchart in figure 1.

A total of 21 studies met inclusion criteria and were included in this review.^1^^,^^5^^,^^9^^,^^22–39^Table 1 provides a brief overview of study findings and their methodological characteristics. 15 of the 21 studies included in the review were published since 2012, with 6 studies published in addiction journals,^9^^,^^24^^,^^27^^,^^32^^,^^37^^,^^39^ 8 in more generic health journals^1^^,^^5^^,^^29–31^^,^^33^^,^^34^^,^^36^ and 7 in other journals.^22^^,^^23^^,^^25^^,^^26^^,^^28^^,^^35^^,^^38^ The reports by Jones et al.^22^^,^^23^ used identical text in parts, discussed the same themes and included data on five spirits producers in both reports.

**Table 1:** Details of included studies and their methodological features

Authors and reference	Publication year	Overview of study and its findings	Methodological characteristics
Grant M^24^	1984	This is a survey of the contents and audiences of educational initiatives sponsored by trade associations prior to the emergence of separate social aspects organizations. Most initiatives targeted young people and focused on moderation. The author underlined the lack of independent evaluation studies of these initiatives, and suggested possibilities for collaboration between the industry and the public health community. (The author became the founding President of ICAP in 1995.)	Of 50 trade associations contacted in 21 countries, 13 confirmed they had sponsored educational initiatives and provided some data and 15 others replied to state that they were not involved in educational initiatives. A narrative summary of both the 13 and 15 respondents is provided. The study was framed as a review rather than a survey. There is no dedicated methods section and limited adherence to conventions in reporting research studies.
Houghton E^9^	1998	This is an update of the survey of education conducted by Grant in 1984 and a comparison of the situation in 1996 by an ICAP author. It included social aspects organizations as well as trade associations. Author findings reported that these initiatives have increased, with some progress on independent evaluations. Drinking driving, youth, moderation and responsibility remain core contents. The author noted that public–private interactions increased.	Of 88 trade associations and social aspects organizations contacted in 33 countries, 32 confirmed they had sponsored educational initiatives [including 90% (*n*=unreported) of social aspects organizations] and provided some data and 27 others replied to state that they were not involved in educational initiatives. A narrative summary of both the 32 and 27 respondents is provided. There is a dedicated methods section and a separate discussion section so that this report more closely resembles a research report than a discussion paper.
Griffin JJ, James W^25^	2006	This study focused on CSR and the relations between six large beer producer companies (three predominantly US-based and three non-US) and their stakeholders from a business studies perspective. Seven types of CSR (philanthropic activity, social reports, business- and social-related community involvement activities, volunteerism, employer matching programmes and charitable foundations) directed at stakeholders were identified. This study described differences among the companies in the types of CSR programmes and identified significant managerial discretion in decision-making about CSR. It interpreted findings that for all firms much activity is investor targeted (but not exclusively so).	The study was designed to address theoretically generated research questions from a business studies perspective. Multiple data sources were used, with data gathered on companies over a two-year period in what might be described as a comparative case study. Similarities and differences between companies is the key basis of inference generation. This was a preliminary study that identifies many variables potentially relevant to CSR.
Smith SW, Atkin CK, Roznowski J^26^	2006	This study reported on a study of ‘responsible drinking’ television advertizements from two large US beer companies shown to teenagers and young adults. Strategically ambiguous messages designed to engender diverse interpretations between varied audience segments were identified. The study concluded that ‘seemingly prohealth messages can serve to subtly advance both industry sales and public relations interests’.	This marketing/communications study was undertaken with a sample size *n*=326 using basic quantitative data for measurement purposes. Four pre-specified hypotheses were tested and support for all four is found. The use of inferential statistics is limited. Findings of hypothesized differences in line with predictions among sub-groups, including by age, suggest that this is a well-developed study, notwithstanding identified limitations.
Kiukas V, Mikkonen J^27^	2009	This is a study of an alcohol education initiative developed as a media campaign by an industry actor in Finland. It critiqued the industry actor’s own evaluation and posited an interpretation of the self-serving nature of the campaign with public opinion hardening against alcohol following increases in consumption and problems following early tax reductions.	This could be interpreted as a form of case study, drawing on some international comparisons and other literature-based data, though may be more appropriately viewed as a commentary piece responding to an important development than a research report. There is little dedicated methodological content other than drawing on data sources, and little adherence to the format of scientific reporting otherwise.
Carah N, Van Horan A^28^	2011	This study reported on the communication activities of DrinkWise. The authors identified that DrinkWise framed alcohol problems as a cultural issue and focused on under-age drinking rather than harmful drinking at the population level. DrinkWise proposes information and education and emphasizes parental and personal responsibility in ways which facilitate the commercial objectives of the alcohol industry.	This a media studies case study of Drinkwise which examined 29 DrinkWise media releases, and matched them to 55 news articles. Theory-based framing analyses were undertaken by two coders. Detailed findings on framing are presented and discussed.
Jernigan DH^29^	2012	This study sought to document the activities of ICAP. Strategies identified ‘include producing scholarly publications with incomplete, distorted views of the science underlying alcohol policies; pressuring national and international governmental institutions; and encouraging collaboration of public health researchers with alcohol industry–funded organizations and researchers’.	This case study drew upon a large array of ICAP and other documentary data sources over an extended period of time to offer a detailed account of the formation and subsequent development of ICAP, and an analysis of its apparent function to counter WHO throughout its history. This is the seminal study of ICAP which has delivered influential insights on industry political strategies, and potentially also into CSR more broadly. There is no dedicated methods section, however, and the nature of the report precludes direct assessment of study limitations.
Pantani D, Sparks R, Sanchez Z, Pinsky I^30^	2012	This study examined the ‘responsible drinking’ initiatives of several alcohol industry actors in Brazil, investigating possible differences between national and transnational companies. The initiatives were largely focused on server training, drink driving and under-age drinking and mostly undertaken by transnational companies using television for informational content.	The study involved content analyses of semi-structured interviews with nine key informants from 5/6 largest companies and 71 opportunistically gathered company documents. Detailed analytic procedures are described, including team involvement in coding data. Triangulation is discussed. This exploratory study yielded useful data from both documentary data sources and the interviews, where direct quotations were used.
Babor TF, Robaina K^1^	2013	This is a study that examined how the CSR activities of the alcohol industry are relevant to public health research community and the science/policy interface. Key areas studied include sponsorship of scientific research, efforts to influence public perceptions of research, dissemination of scientific information and related industry-funded policy initiatives. The authors concluded that the industry intensified its activities in this area.	This is a narrative review of peer-reviewed data supplemented with grey literature data sources and reviews of the alcohol policy literature. This study provides seminal data on CSR and science. There is no dedicated methods section, however, and the nature of the report precludes direct assessment of study limitations.
Jones P, Hillier D, Comfort D^22^	2013	This paper provides an exploratory examination of the CSR reports of the 10 biggest spirits producers. It identified how corporate actors focus on responsible drinking, and also addressed other issues such as environmental issues and impacts within the workplace. Four broad themes are discussed characterizing the reports as a whole, eschewing any examination of differences between companies.	This study was conceived within a business studies/management framework including ideas about the nature of CSR in that literature. The data sources are identified, though there is limited information on data collection timeframes and no content on how data were analyzed, and thus how the reported themes were developed.
Jones P, Hillier D, Comfort D^23^	2013	This paper is very similar to the previous one except that it discussed the CSR of the five largest spirits (as included above) and five largest beer producers. The same four broad themes are discussed.	See comments made above.
Yoon S, Lam TH^5^	2013	This study assessed the CSR initiatives of three largest transnational alcohol producers, as well as ICAP. The three main findings were that CSR was used (i) to frame alcohol-related issues as a matter of personal responsibility and thus shift attention away from the industry; (ii) to promote voluntary/self-regulation in order to prevent or delay regulation and (iii) as an indirect form of marketing.	Detailed information is provided on data collection and analysis. Data sources included the websites of the organizations, social media platforms, media reports and details of search methods and outcomes and eligibility criteria are presented. Total of 281 included documents were coded in a process involving both authors and thematically analyzed. Detailed content is reported for each of the main findings.
Lyness SM, McCambridge J^31^	2014	This is a study of charities funded by UK alcohol industry actors and of their involvement in alcohol policy making, and thus potential to be used for political influence. It found that five charities received funding from the industry and were active in alcohol policy, two of whom had a distinct position from other health NGOs during a period of policy controversy.	Detailed information is provided on data collection. Data sources included the websites of the charities regulators for England and Scotland organizations and the charities own websites. Details of search methods and outcomes, and eligibility criteria are presented. Both authors were involved in the analysis and detailed findings are reported.
McCambridge J, Kypri K, Miller P, Hawkins B, Hastings G^32^	2014	This paper treated Drinkaware, a UK social aspects organization, as a case study. It examined Drinkaware’s activities in the context of the literature on other such organizations and explored concerns about this organization and UK alcohol policy.	This paper does not have a methods section or present details of information on data collection and analysis. The absence of such information precludes direct assessment of study limitations, e.g. which data sources may have been missed.
Esser MB, Bao J, Jernigan DH, Hyder AA^33^	2016	This study examined the evidence base for drink driving initiatives sponsored by the alcohol industry. Social aspects organizations and trade associations sponsored more of these initiatives than any individual company (41% combined). About <1% of these initiatives were consistent with evidence of effectiveness in reducing drink driving.	This study used an IARD dataset of global initiatives and extracted and coded data according to a detailed content analysis protocol. Two authors were involved in coding including potential for harm, marketing and policy influence. Basic quantitative data are provided to enable comparisons between regions, actors and other variables of interest, as well as presenting the overall picture and drawing clear conclusions.
Herrick C^34^	2016	This paper provides a discussion about public–private partnerships involving public health and alcohol industry interests and the ways in which CSR represents a key site of conflict. It used a specific example a South African initiative sponsored by a global producer.	This could be interpreted as a form of case study, presenting contextual data on the UN Sustainable Development Goals and other material, though may be more appropriately viewed as a well-considered thinkpiece on important issues rather than a research report. There is no dedicated methodological content.
Jones SC, Wyatt A, Daube M^35^	2016	This is a study of corporate social marketing in the form of the promotion of responsible drinking as a means of CSRs. It identified that such initiatives (i) contain ambiguous messages; (ii) are not effective in reducing alcohol drinking but could improve the public perceptions of the industry; (iii) shift attention away from effective policies to reduce harmful drinking.	This literature review examined disparate sources on corporate social marketing and alcohol and presents coherent arguments in the interpretation of the literature. There is no dedicated methods section, so the adequacy of data collection and forms of analysis used are unknown. As such, this could also be viewed as a discussion paper which effectively pulls together relevant literatures.
Petticrew M, Fitzgerald N, Durand MA, Knai C, Davoren M, Perry I^36^	2016	This study analyzed a responsible drinking campaign by Diageo in Ireland with a view to investigation of framing. It found that (i) alcohol issues were presented as behavioural rather than health problems; (ii) there was a focus on public opinion rather than scientific evidence; (iii) the preferred solutions were information provision and education.	Documentary analyses of campaign materials used newspapers, media interviews, websites and social media data sources. All data were coded inductively by two researchers among the team to produce a thematic analysis. Detailed presentation of qualitative findings attests to the rigour of the analytic processes.
Pettigrew S, Biagioni N, Daube M, Stafford J, Jones SC, Chikritzh T^37^	2016	This study of a responsible drinking campaign by the Australian social aspects organization DrinkWise took advantage of commercial and health communications methods to reverse engineer the content i.e. identify retrospectively how message content may have been strategically developed. It found that heavy drinkers were unable to relate to the heavy drinkers portrayed in the ads, and, therefore, could not see the need to modify their own drinking behaviours.	This study used introspection data, a consumer research technique to gather reflections on open questions communicated by e-mail from a target population. The research aims were elaborated within an advertizing effects conceptual framework which guided coding procedures. A thematic analysis examined messages processing pathway and data are presented which extend to perceived behavioural outcomes.
Pietracatella RJ, Brady D^38^	2016	This is a case study of the media content of DrinkWise, the Australian social aspects organization. Key findings were that DrinkWise engaged in ‘insulating the alcohol industry against evidence-based public health harm reduction strategies, by engaging in agenda building through industry-friendly framing of the alcohol issue, and dissemination of information subsidies to elites and policy-makers’.	This study is conceptualized within a media/public relations and used both a content analysis and rhetorical framing analysis to examine 40 and 58 media releases respectively. The former examined the detailed content of the messages and their target audiences whilst the latter used theoretically based analytic methods to identify definition of the alcohol problem and possible solutions favoured by industry actors.
Pantani D, Peltzer R, Cremonte M, Robaina K, Babor T, Pinsky I^39^	2017	This study examined the marketing potential of alcohol industry CSR initiatives in Latin America and the Caribbean. More than half of these initiatives had the potential to promote a brand or a product, and most were contrary to recommendations made by the WHO to reduce harmful drinking.	This study used an IARD dataset of industry initiatives and extracts, and coded data according to a detailed protocol. A team of nine expert coders were involved in coding and inter-rater reliability was examined and reported. Statistical testing was used to evaluate differences between variables and selected qualitative data is presented to illustrate the nature of the potential for marketing.

As this review did not select studies for inclusion by methodological characteristics, there is great heterogeneity among the 21 studies in study design and, consequently, in risks of bias, making any formal assessment of risk of bias redundant. It will be seen from table 1 that many existing studies of alcohol industry CSR have weak study designs and are thus not capable of producing robust findings. Among the existing studies which are more theoretically informed and address well-constructed hypotheses, findings are largely characterized by their authors as being preliminary or exploratory. The literature as a whole cannot thus be characterized as being well developed in methodological terms, carrying important implications for the interpretation of the study findings presented here.

### Types of alcohol industry CSR initiatives

We identified five main types of CSR initiative undertaken by alcohol industry actors: alcohol information and education provision; drink driving prevention; research involvement; policy involvement and the creation of social aspects organizations (see table 2). These categories are not mutually exclusive; drinking driving prevention, e.g. is largely a sub-category of information and education, though does also involve other interventions. The creation of social aspects organizations is also a discrete type of CSR, rather than being an organizational vehicle for the performance of other types of initiatives. In addition to CSR initiatives directed at reducing harmful drinking, several studies discussed philanthropy in relation to non-alcohol issues such as emergency humanitarian aid, arts and culture. These initiatives included, e.g. the provision of drinking water,^23^ the building of schools^22^ and support to disaster relief charities.^5^

**Table 2:** Typology of alcohol industry CSR initiatives

Type of CSR initiatives	Description	Detailed content and implementation methods
Alcohol information and education provision	Provision of education and information (on issues such as personal and/or parental responsibility, moderation, under-age drinking, health effects of drinking alcohol) including in campaigns	• Mass media: television and radio commercials,^24^^,^^26^^,^^27^^,^^30^^,^^39^ videos,^36^^,^^39^ cinema commercials,^24^^,^^26^^,^^27^ newspapers,^28^^,^^36^ billboards, on premise, cinema advertizing and transit advertizing^35^ • Web-based sources: website, mobile devices, social media and online advertizement^5^^,^^35–38^ • Provision at point of sale^39^ • Merchandising material^39^ • Gift packs and tips to school leavers in airports^35^ • Peer group activities^24^ • Information for professionals: medical education resources, specialist symposia^24^ • Schools^1^^,^^9^ • Interventions in bars/taverns: workshops and educational interventions^34^ • Other documents: information kit, booklet, teacher’s handbook, parents’ booklets, posters, blood alcohol estimation cards, display cards and audio-visual resources^24^
Drink driving prevention	Interventions for drink driving prevention including information and education	• Mass media^33^: television and radio commercials,^26^^,^^30^^,^^39^ labels, banners^30^ • Education in discos^9^ • Electronic simulator^9^ • Videos^9^^,^^25^^,^^39^ • Driving experiment^9^^,^^33^ • Promotion at point-of-purchase and workplaces^30^ • Merchandising material^30^ • Dissemination of documents: kits and reports,^30^ leaflets, posters, stickers and other handouts and stickers with legal age enforcement^33^ • Free ride programmes^1^^,^^33^^,^^39^ • Sobriety checkpoints^33^ • Ignition interlocks^33^ • Blood alcohol concentration tests for young drivers^33^ • Print and video materials, mobile app^39^ • Taxi ride discount^39^ • Breathalyzer donation^30^^,^^39^
Research involvement	Support of research/researchers and dissemination of research findings on alcohol and health issues	• Hosting scientific conferences^1^^,^^24^^,^^29^ • Formulation of the ‘Dublin Principles of Cooperation’ on industry-researcher collaborations^29^ • Publication and dissemination of scientific documents: essays, monographs, reports, briefing papers, peer-reviewed journal articles, special issues in scientific journals^1^^,^^24^^,^^29^ and public opinion surveys^33^ • Support for grant making organizations^1^ • Funding researchers and support of research centres^1^ • Support of researchers to provide briefings to journalists^1^ • Funding critiques written by paid academics^1^
Policy involvement	Activities designed to influence policy making	• Publication and dissemination of policy documents: reviews of alcohol policy issues, charters, working papers, guides to policy implementation and policy tool kits^29^ • Eexpert technical assistance in implementing alcohol policies for less-resourced countries^1^^,^^29^ • Participation in high level meetings of policy making organizations^29^ • Dissemination of publications at government consultations^1^ • Support to charities that are active in alcohol policy, or that have board members from the alcohol industry (which are active in alcohol policy)^31^
Creation of social aspects organizations	Development and funding of social aspects organizations	See Supplementary material S2 for detailed content

Table 3 presents data on information and education provision and drink driving prevention, the two types of CSR initiatives employed by individual alcohol companies that were examined in the different studies included within this review. There were no studies of the CSR initiatives of individual companies and their involvement in research or policy making, as these activities appear largely undertaken by social aspects organizations, trade associations or third parties.

**Table 3:** Identification of individual alcohol companies’ involvement in two types of CSR initiatives

	Types of CSR initiatives supported by the industry actor, as studied in the literature		Geographical coverage (alphabetical order)
	Alcohol information and education provision	Drink driving prevention	
ABInBev^a,1,5,23,25,26,30,33,39^	X	X	Argentina Brazil Bolivia China Germany Paraguay Uruguay USA
Bacardi^23^	X	–	Not specified
Brown-Forman^23^	X	–	USA
Carlsberg^33^	–	X	Sweden
Diageo^22^^,^^23^^,^^25^^,^^30^^,^^31^^,^^33^^,^^36^^,^^39^	X	X	Brazil China Columbia Ghana Ireland Uruguay USA
FEMSA^30^	–	X	Brazil
Florida Bebida^39^	–	X	Costa Rica
Heineken^25^^,^^39^	X	X	Africa Brazil
Kirin Brazil (formerly known as Schincariol)^a,30^	X	X	Brazil
Molson Coors^a,25,26^	X	X	USA
Pernod Ricard^5^^,^^22^^,^^23^	X	–	India Poland Thailand
SABMiller ^a^^,b,1,5,23,25,33,34^	X	X	‘developing world’ San Salvador South Africa
Thai Beverage^22^	X	–	Thailand
Tsingtao^23^	–	–	China
Williams Grant and Sons^22^	X	–	Not specified

a The name of the industry actors may differ in the different publications included in this review, and, in this manuscript, we use the official name of the actor as of September 2017.

b SABMiller was bought out by ABInBev in 2016 and does not operate under this name any more, so we used the latest name of the company, before its acquisition, in this review.

It appears from table 3 that many CSR initiatives that have been studied were implemented in low and middle-income countries (LMICs; as classified by the World Bank).^5^^,^^22^^,^^23^^,^^25^^,^^30^^,^^34^^,^^39^ Some CSR initiatives were implemented in the USA^23^^,^^25^^,^^26^ and in Europe.^5^^,^^33^^,^^36^ The vast majority of these initiatives targeted young people and their parents.^5^^,^^22^^,^^23^^,^^25^^,^^26^^,^^30^^,^^33^^,^^36^^,^^39^ Among partners identified were schools,^9^ hospitals,^9^ universities and researchers,^1^^,^^9^^,^^29^ international and national drugs and/or addiction agencies,^29^^,^^34^ traffic safety agencies,^9^ automobile companies,^9^ public relations companies,^5^^,^^36^ sport teams or players^30^^,^^39^ and charities.^9^^,^^34^^,^^39^

The social aspects organizations identified in included studies are presented in Supplementary material S2. There was most extensive study of DrinkWise (Australia). In contrast to CSR initiatives by individual alcohol companies, social aspects organizations that have been studied were mostly based and operating in high-income countries, particularly in Europe and North America.^9^^,^^24^^,^^27^^,^^28^^,^^31^^,^^32^^,^^35^^,^^37^^,^^38^ The International Center for Alcohol Policies (ICAP) was also involved in research and policy activities in LMICs.^29^ CSR initiatives targeted academics and health professionals, governments, with whom they established partnerships,^1^^,^^29^^,^^31^^,^^32^^,^^38^ as well as directly targeting young people and their parents.^9^^,^^27^^,^^28^^,^^35^^,^^37^^,^^38^

### Evidence of the effects of CSR initiatives on harmful drinking

We found little evidence supportive of the possibility that alcohol industry CSR initiatives may benefit public health.^26^^,^^27^^,^^32–35^^,^^39^ This concern was first articulated by Grant more than 30 years ago,^24^ and according to Babor and Robaina;^1^

*‘the global initiatives promoted by the alcohol industry are overwhelmingly derived from approaches of* unknown *or minimal effectiveness or approaches shown to be ineffective through systematic scientific research. Moreover, the industry initiatives only rarely include practices that the WHO [World Health Organization] and the public health community consider to have good evidence of effectiveness, and few have been evaluated in the low- and middle income countries where they are now being disseminated’.*^1^

For drink driving prevention specifically, in a major study Esser *et al.*^33^ analyzed 266 CSR activities undertaken by the alcohol industry. They found that whilst alcohol industry reported an evaluation of their initiatives in over one-third of cases, only 3% of these evaluations measured outcomes to establish effectiveness in reducing drink driving, and none of these were rigorous studies.^33^ About <1% of studied initiatives were based on any scientific evidence of effectiveness for reducing drink driving.^33^ More than two-thirds of the actions examined in this study were rated as having potential for harm, 88% potential for marketing and 7% potential for policy influence.^33^

Pantani *et al.*^39^ reported that the majority of CSR initiatives undertaken by alcohol industry actors in Latin America and the Caribbean were likely to promote brands or products. Herrick^34^ referred to mixed findings of an evaluation of a specific community based prevention initiative in South Africa, without presenting the data. McCambridge *et al.*^32^ and Jones *et al.*^35^ discussed evaluations conducted by industry actors, or by third parties which had relations with the alcohol industry, and which involved market research rather than scientific studies of effectiveness in reducing harmful drinking. The papers by Jones *et al.*^22^^,^^23^ underline the business interests underpinning CSR activities, rather than potential societal or public health benefit, as does the study by Griffin and James.^25^

Rather than having positive effects in reducing harmful drinking, a number of studies provide data to suggest that alcohol industry CSR initiatives may have the opposite effects. Pettigrew *et al.*^37^ found; *‘the “How to Drink Properly” advertisement [by Drinkwise Australia] was liked and appeared to reinforce current drinking attitudes and behaviours, especially the idea that alcohol consumption is a natural element of social occasions*’ and was ‘*supportive of existing social norms relating to heavy drinking among members of this age group*’. Smith *et al.*^26^ similarly found that young people shown ‘responsible drinking’ messages viewed them as glamorizing alcohol consumption among young adults, and as being similar to marketing. They further demonstrated that the sophisticated content was designed to segment audiences and engender different interpretations of the same contents. Pantani *et al.*^39^ found that more than half of the initiatives developed by industry actors participating in the ‘Producers’ Commitments’ in Latin America and the Caribbean had marketing potential. Yoon and Lam^5^ identified that philanthropy yields benefits to corporate donors in terms of social media and website usage and that ‘brand stretching’ is involved in many activities.

### Political uses of CSR by alcohol industry actors

Several studies examined the political uses of CSR, and the extent to which they furthered the strategic goals of industry actors, with implications for harmful drinking. Yoon and Lam^5^ provided a good illustration of a company’s use of CSR for economic and political purposes. SABMiller’s sustainable development initiatives in Africa were explicitly linked by the company to their business interests as follows; *‘The business objectives for engaging with small-scale farmers have differed from region to region, ranging from strengthening government relations to securing future input supplies’.*^5^^,^^40^

Jernigan reported on the extensive efforts made by ICAP to oppose alcohol policies motivated by public health considerations;^29^*‘Much of the ICAP’s activities have focused on countering the inﬂuence of the WHO* […]. [ICAP] *provided model national and global alcohol policies based on the least effective strategies, and offered technical assistance in how to adopt and implement these policies* […] *they excluded or attempted to refute evidence regarding the most effective strategies to reduce and prevent alcohol-related harm’.*^29^

Jernigan^29^ gives one example of the success of these efforts: ICAP claimed that it was able to change the terms ‘alcohol and other drugs’ used by the US Center for Substance Abuse Prevention, which, from the alcohol industry’s point of view, ‘*incorrectly and unjustly equates our products with illegal drugs*’, and replaced it with the less problematic expression ‘substance abuse’.^29^ Jernigan provides internal tobacco company documentary evidence resulting from ownership of a major alcohol producer that ICAP was established as a form of CSR specifically designed for the purposes of policy influence.^29^

Similarly, several studies examine how CSR is used to frame alcohol issues and to focus on responsible alcohol drinking;^5^^,^^28^^,^^32^^,^^36^^,^^38^ ‘*shifting the attention from those who manufacture and promote the products to those who consume them*’, as suggested by Yoon and Lam.^5^ They also identify how well CSR lends itself to making arguments in favour of self-regulation. Other authors noted that the alcohol industry often cited ‘culture’ as a contributor to alcohol issues, whilst avoiding any discussion on the role that the industry itself has in shaping that drinking culture.^28^^,^^36^^,^^38^ Carah and Van Horen^28^ identify how the Australian social aspects organization Drinkwise worked with journalists and the media to frame alcohol issues in the ways sought by industry actors, providing source material in media releases that is converted into news articles. Framing also has direct political implications, as identified by Pietracatella and Brady;^38^ ‘*through the PR tools of issue framing and information subsidies, DrinkWise media relations functions as indirect lobbying to policy-makers and elites on behalf of alcohol industry interests’*.

In their study of a Diageo information and education campaign, Petticrew *et al.*^36^ identified; ‘*indirect lobbying in order to oppose public health measures by creating front groups, and by forming alliances with civil society organisations and consumers; the promotion of non-regulatory initiatives; a focus on individual responsibility, and the (mis)behaviour of a small minority; the omission of “health” from discussions; and misrepresentation of the evidence base*’. McCambridge *et al.*^32^ noted the high profile support Drinkaware has obtained from successive UK governments and the legitimacy this provides to use of their materials within the National Health Service. Lyness and McCambridge^31^ identified that alcohol industry actors appear to be funding charities to influence policy, as the charities they fund adopt different positions within policy making to other alcohol charities.

## Discussion

This study describes a literature that is early in its development, and has been growing rapidly, with three-quarters of included studies having been published since 2012.^1^^,^^5^^,^^22^^,^^23^^,^^29^^–^^39^ Given the clear significance to global health of the issues addressed by this literature, it can be anticipated that it will continue to grow quickly. This is the first systematic review of alcohol industry CSR. Future studies of alcohol industry CSR may be informed by the methodological limitations of the existing literature.

This review uses systematic methods for data collection and only peer-reviewed papers were included here, thus enhancing the validity of the findings. Review-level limitations are also inherent in this study design. On the one hand, the exclusion of CSR content that is explicitly integrated with marketing content, or which is concerned with core roles as economic operators (such as server training or reducing the alcohol content of products), presents a narrow view of CSR. This is especially important in light of the findings of Pantani *et al.*^39^ which emphasize synergies between marketing and CSR. It is thus likely that CSR studies not included here because they are concerned with CSR initiatives which are integrated with marketing content (see e.g.^20^) will deepen our understanding of alcohol industry CSR, and particularly its instrumental uses. It remains possible that some CSR initiatives which have not been studied here may contribute towards reductions in harmful drinking. On the other hand, the exclusion of CSR initiatives closely linked to core business practices facilitates interrogation of the claims made about possible impacts on harmful drinking from initiatives that are declared to have this aspiration as their sole purpose.

The inclusion only of studies published in English means that we may have missed studies published in other languages (though none were identified in the searches). Nonetheless the absence of formal risk of bias assessment constitutes a limitation of this review. Other possible limitations relating to data collection, rather than analysis, concern the possibility of having missed studies of the CSR activities of trade associations or small companies, and initiatives at regional levels in federal political systems. Perhaps the most important limitation stems from the ‘apples and oranges’ nature of this review. As the first evidence synthesis in this area, we placed no restrictions on study characteristics warranting inclusion (other than presentation of research data in peer-reviewed journals reports). Approximately half the included studies lack methodological details that permit any meaningful assessment of risk of bias, other than to assess their reporting as weak. The study designs are also highly variable, and largely exploratory in nature, meaning that caution should be exercised in making inferences about this research literature as a whole, other than to interpret it as being early in its development and in some ways more useful for hypotheses generation rather than for testing.

Consequently, the remainder of this discussion will mirror the narrative presentation of results, and primarily focus on those studies without what are regarded as the most important methodological limitations.^5^^,^^25^^,^^26^^,^^28^^,^^30^^,^^31^^,^^33^^,^^36^^,^^37^^,^^39^

We now make a number of observations about what can be inferred as being securely known at this stage in the evolution of this literature, and what remains to be established in further research. There is no robust evidence that alcohol industry CSR initiatives reduces harmful drinking. A brief examination of the websites (or CSR reports) of major alcohol companies and of more than 40 social aspects organizations in existence worldwide will indicate how limited is the coverage of existing studies. It is judged highly unlikely, however, given the nature of the data available in existing well-designed studies of the effects of CSR, that further studies will alter this main finding. The study by Esser et al. on drink driving^33^ is particularly strong in this respect. This study drew on an extensive global database of CSR activities compiled by the global alcohol producers to demonstrate their contribution to the WHO Global Strategy to Reduce the Harmful Use of Alcohol.^33^ This study, appropriately in our view, reaches such a definitive conclusion on the ineffectiveness of the CSR initiatives studied, that it seems highly unlikely that further investigations would alter the main conclusions drawn by reasonable observers. A number of studies, most notably the study by Pantani et al.^39^ have identified and assessed potential harms arising from alcohol industry CSR. Further conceptual and empirical work on direct and indirect harms will be useful in exploring how far such potential is translated into adverse impacts on harmful drinking.

Beyond the direct effects on harmful drinking, the use of CSR in obtaining access to policy making and/or influencing decision-making, creates potential for indirect effects on harmful drinking. There is a strong convergence of evidence from well-designed studies^5^^,^^26^^,^^28^^,^^36–38^ on the promotion of responsible drinking. This attests to the key importance of framing, and indicates that the framing of alcohol consumption as a matter for individual decision-making has powerful implications within policy making. This framing lies in direct conflict with a public health conceptualization of harmful drinking, and with the scientific evidence-base on how it may be reduced.

The types of CSR initiatives identified here offer a more specific typology in relation to harmful drinking than those identified by Griffin and James.^25^ The identification of the creation of social aspects organizations as a form of CSR appears particularly important in relation to research and policy involvement, where the actions of individual companies have not yet been studied. The study by Lyness and McCambridge^31^ also identifies industry funding of charities to have implications for safeguarding policy making from commercial interests. Social aspects organizations, as with trade associations, appear to represent the interests of the industry as a whole (or key sections of it), hence their identification as social aspects and public relations organizations primarily concerned with managing issues that may arise on behalf of industry interests.^41^ Trade associations have reduced or discontinued their involvements in public information and education, and social aspects organizations now combine this type of CSR initiative with others such as policy involvement, making them distinct actors and potentially key organizers of industry wide CSR. In our review, the only dedicated studies of social aspects organizations are one study of ICAP,^29^ one study of Drinkaware^32^ and four studies of Drinkwise.^28^^,^^35^^,^^37^^,^^38^

## Conclusions

Claims that alcohol industry CSR initiatives reduce harmful drinking have no foundations in scientific evidence. Preliminary evidence from an emerging literature in fact suggests two sets of alternative possibilities are more likely; (i) that alcohol industry CSR initiatives have no direct impacts on rates of harmful drinking, or that any impacts are adverse rather than beneficial; and (ii) that industry CSR initiatives may be used to interfere with the framing of the nature of alcohol-related issues, and thus the development of evidence-informed public policies that can reduce harmful drinking. These conclusions support the further development of political and instrumental theories of CSR. Although this research literature is at an early stage of development, there are clear implications for policy makers arising from this study; both claims about the putative benefits of alcohol industry CSR and industry framing of alcohol issues are problematic and should not be allowed to impede the development of evidence-informed public policies if harmful drinking is to be reduced. There are also clear implications for researchers. There is an urgent need to rectify the under-development of this literature and to develop and apply innovative theories and robust scientific methods to the study of CSR and related aspects of alcohol industry practices. Researchers, research funders, policy makers and other stakeholders should pay careful attention to how far, and in which ways, CSR forms part of the business and political strategies of alcohol industry actors, increasing harmful drinking and blocking the adoption of effective policies.

## Supplementary data

Supplementary data are available at *EURPUB* online.

## Funding

This work was supported by a Wellcome Trust Investigator Award in Humanities and Social Science (200321/Z/15/Z) to JM.

*Conflicts of interest*: The authors declare that they have no competing interest.

**Key points**

- There is no robust evidence that alcohol industry CSR initiatives reduce harmful drinking.
- Alcohol industry CSR initiatives may be used to interfere with the framing of the nature of alcohol-related issues.
- This framing lies in direct conflict with a public health conceptualization of harmful drinking, and with the scientific evidence-base on how it may be reduced.
- The scientific study of alcohol industry CSR is grossly under-developed and research on this subject can play a key role in developing more effective alcohol policies.

## Supplementary Material

Supplementary Material 1

Supplementary Material 2
