# Supplementary material for: Alcohol industry corporate social responsibility initiatives and harmful drinking: a systematic review
Source: Eur J Public Health. 2018 Apr 25;28(4):664–73. doi: 10.1093/eurpub/cky065 (PMC6051456; doi:10.1093/eurpub/cky065)
Supplement: Supplementary Material 1 [file cky065_ejph-2017-12-srm-0972-file003.doc]

Supplementary material 1: Search strategy, searches conducted on 21.7.2017

# Database: Medline (Ovid interface)

1 corporat*.mp. or exp Industry/ or industr*.mp. or compan*.mp. or business*.mp. or firm*.mp.

2 exp Alcohols/ or alcohol*.mp. or drink*.mp. or exp Alcohol Drinking/

3 1 and 2

4 Social Responsibility/ or "social responsibility".mp.

5 3 and 4

6 limit 5 to yr="1980-current"

# Database: Embase (Ovid interface)

Same as Medline

# Database: PsycINFO (Ovid interface)

Same as Medline

# Database: Web of Science Core Collection (WoS interface)

TS=(((corporat* OR industr* OR compan* OR business* OR firm*) AND (alcohol* OR drink*)) AND ("social responsibility"))

Timespan: 1980-2017

# Database: Business Source Premier (EBSCOhost interface)

( (corporat* OR industr* OR compan* OR business* OR firm*) AND (alcohol* OR drink*) ) AND "social responsibility"

AND limit to 1980-present

# Database: CINAHL Plus (EBSCOhost interface)

Same as Business Source Premier

AND limit to 1980-present

# Database: Scopus (Scopus interface)

TITLE-ABS-KEY ( ( ( corporat* OR industr* OR compan* OR business* OR firm* ) AND ( alcohol* OR drink* ) ) AND "social responsibility" ) AND PUBYEAR > 1980
