# Supplementary material for: Alcohol industry corporate social responsibility initiatives and harmful drinking: a systematic review
Source: Eur J Public Health. 2018 Apr 25;28(4):664–73. doi: 10.1093/eurpub/cky065 (PMC6051456; doi:10.1093/eurpub/cky065)
Supplement: Supplementary Material 2 [file cky065_ejph-2017-12-srm-0972-file004.docx]

Supplementary Material 2: Social aspects organisations identified in CSR studies

|  | **Types of CSR initiatives supported by the industry actor as studied** | | | |
| --- | --- | --- | --- | --- |
|  | Alcohol information and education provision | Drink driving prevention | Research involvement | Policy involvement |
| **ABMRF/The Foundation for Alcohol Research (USA)** (1) |  |  | X |  |
| **Asociación de Distribuidores de Grandes Marcas (ADIGRAM) (Spain)** (2) | X |  |  |  |
| **Avec Moderation (formerly know as Entreprise et Prevention) (France)** (2) | X |  |  |  |
| **Beer Institute (USA)** (2) | X |  |  |  |
| **Belgium Arnoldus Group (Belgian Brewers Association)** (2) | X | X |  |  |
| **Brewers Association of Australia and New Zealand Inc. (formely known as Australia Associated Brewers and the Australia Brewers Foundation)** (2) | X | X |  |  |
| **DIFA Forum (Germany)** (2) | X | X |  |  |
| **Drinkaware (UK)** (3, 4) | X |  |  | X |
| **DrinkWise (Australia)** (4-8) | X |  | X |  |
| **European Foundation for Alcohol Research (ERAB) (Belgium)** (1) |  |  | X |  |
| **FORUM pour la consommation responsable des boissons alcoolisées (Belgium)** (2) | X |  |  |  |
| **Foundation for Advancing Alcohol Responsibility (formerly known as the Century Council) (USA)** (2) | X | X |  |  |
| **Foundation for Responsible Alcohol Consumption (Stichting Verantwoorde Alcoholconsumptie STIVA) (Netherlands)** (2) | X |  |  |  |
| **GODA Gode Alkoholdninger (Denmark)** (2) | X | X |  |  |
| **Industry Association for Responsible Alcohol Use (ARA) (South Africa)** (2) | X |  |  |  |
| **Institut de Recherches Scientifiques Economiques et Sociales sur les Boissons (IREB) (France)** (1, 9) |  |  | X |  |
| **International Centre for Alcohol Policies (ICAP)** (1, 10-12) ^1^ | X | X | X | X |
| **International Scientific Forum on Alcohol Research (ISFAR) (USA)** (1) |  |  | X |  |
| **Portman Group (UK)** (1, 2, 13) | X |  | X | X |
| **Worldwide Brewing Alliance (UK)** (1) |  |  | X |  |

^1^ In 2014, ICAP merged with the Global Brewers Group to form IARD (14).

1. Babor TF, Robaina K. Public health, academic medicine, and the alcohol industry's corporate social responsibility activities. American Journal of Public Health. 2013;103(2):206-14.

2. Houghton E. A comparative analysis of alcohol education programs sponsored by the beverage alcohol industry. Journal of Alcohol and Drug Education. 1998;43(3):15-33.

3. Lyness SM, McCambridge J. The alcohol industry, charities and policy influence in the UK. European Journal of Public Health. 2014;24(4):557-61.

4. McCambridge J, Kypri K, Miller P, Hawkins B, Hastings G. Be aware of Drinkaware. Addiction. 2014;109(4):519-24.

5. Jones SC, Wyatt A, Daube M. Smokescreens and Beer Goggles: How Alcohol Industry CSM Protects the Industry. Soc Mark Q. 2016;22(4):264-79.

6. Pietracatella RJ, Brady D. Drink wisely Australians, but keep drinking: does the alcohol industry in Australia frame the discourse on alcohol to maintain hegemony? Media International Australia. 2016(160):53-66.

7. Pettigrew S, Biagioni N, Daube M, Stafford J, Jones SC, Chikritzhs T. Reverse engineering a ‘responsible drinking’ campaign to assess strategic intent. Addiction. 2016;111(6):1107-13.

8. Carah N, van Horan A. Drinkwise, enjoy responsibly: News frames, branding and alcohol. Media International Australia. 2011(141):5-16.

9. Grant M. The Moderating Influence: A Review of Trade-Sponsored Alcohol Education Programmes. British Journal of Addiction. 1984;79(4):275-82.

10. Yoon S, Lam T-H. The illusion of righteousness: corporate social responsibility practices of the alcohol industry. BMC Public Health. 2013;13(1):630.

11. Esser MB, Bao J, Jernigan DH, Hyder AA. Evaluation of the Evidence Base for the Alcohol Industry’s Actions to Reduce Drink Driving Globally. American Journal of Public Health. 2016;106(4):707-13.

12. Jernigan DH. Global alcohol producers, science, and policy: the case of the International Center for Alcohol Policies. American Journal of Public Health. 2012;102(1):80-9.

13. Kiukas V, Mikkonen J. The Finnish brewing industry and alcohol education. NAT Nordisk alkohol & narkotikatidskrift. 2009;26(4):456-61.

14. Market wired. World's Leading Producers of Beer, Wine, and Spirits Launch International Alliance for Responsible Drinking (IARD) <http://www.marketwired.com/press-release/worlds-leading-producers-beer-wine-spirits-launch-international-alliance-responsible-1957665.htm>. Accessed [October 2017]
